# Manipulating plant phylogenetic diversity for green roof ecosystem service delivery

**DOI:** 10.1111/eva.12703

**Published:** 2018-09-28

**Authors:** J. Scott MacIvor, Nicholas Sookhan, Carlos A. Arnillas, Anushree Bhatt, Shameek Das, Simone‐Louise E. Yasui, Garland Xie, Marc W. Cadotte

**Affiliations:** ^1^ Department of Biological Sciences University of Toronto Scarborough Toronto Ontario Canada; ^2^ Department of Ecology and Evolutionary Biology University of Toronto Toronto Ontario Canada; ^3^ Department of Physical and Environmental Sciences University of Toronto Scarborough Toronto Ontario Canada; ^4^ School of Earth, Environmental, and Biological Sciences Queensland University of Technology Brisbane Queensland Australia; ^5^ Department of Biology Saint Mary's University Halifax Nova Scotia Canada

**Keywords:** biodiversity, building cooling, ecophylogenetics, ecosystem function, green infrastructure, plant selection, stormwater capture

## Abstract

Plant species and functional trait diversity have each been shown to improve green roof services. Species and trait differences that contribute to ecosystem services are the product of past evolutionary change and phylogenetic diversity (PD), which quantifies the relatedness among species within a community. In this study, we present an experimental framework to assess the contribution of plant community PD for green roof ecosystem service delivery, and data from one season that support our hypotheses that PD would be positively correlated with two services: building cooling and rainwater management. Using 28 plant species in 12 families, we created six community combinations with different levels of PD. Each of these communities was replicated at eight green roofs along an elevation gradient, as well as a ground level control. We found that the minimum and mean roof temperature decreased with increasing PD in the plant community. Increasing PD also led to an increase in the volume of rainwater captured, but not the proportion of water lost via evapotranspiration 48 hr following the rain event. Our findings suggest that considering these evolutionary relationships could improve functioning of green infrastructure and we recommend that understanding how to make PD (and other measures of diversity) serviceable for plant selection by practitioners will improve the effectiveness of design and ecosystem service delivery. Lastly, since no two green roof sites are the same and can vary tremendously in microclimate conditions, our study illustrates the importance of including multiple independent sites in studies of green roof performance.

## 1 INTRODUCTION

**Figure 1:**
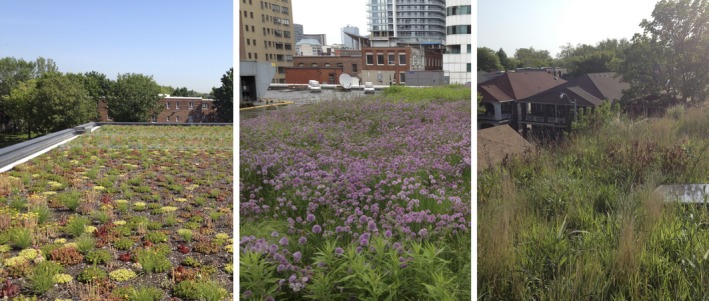
Three extensive green roofs all from Toronto, Canada (from left to right): Regent Park swimming pool, Mountain Equipment Co‐op, a residential home

Green roofs are becoming increasing common in cities because they help alleviate the negative effects of impervious surfaces on building rooftops (Gaffin, Rosenzweig, & Kong, 2012; Gill, Handley, Ennos, & Pauleit, 2007) (Figure 1). They are comprised of plants and substrate on top of conventional roofs and are promoted primarily for their role in cooling buildings in warm seasons, and capturing rainwater (Getter & Rowe, 2006; Oberndorfer et al., 2007; Tzoulas et al., 2007). Building cooling is the result of several mechanisms that relate to the green roof plant community, including species type and vegetative cover, the evapotranspiration of water, reflectivity and absorption of solar radiation, shading, and trapping air pockets in the plant canopy architecture that insulate (Del Barrio, 1998; MacIvor, Margolis, Perotto, & Drake, 2016). These effects reduce the total amount of energy required to regulate building temperatures (Eumorfopoulou & Aravantinos, 1998; Wong, Chen, Ong, & Sia, 2003). Green roof vegetation and substrate also capture rainwater as it permeates the growing substrate—facilitated by plant root penetration, thereby reducing the total volume of water runoff and the peak flow rate of water during and immediately after a rain event. This water is used by the plants for their metabolism and released to the atmosphere via evapotranspiration, which increases the capacity of a green roof to capture more rain after the next storm event. Overall, this reduces the total amount of water that reaches local sewage treatment facilities and, additionally, helps reduce erosion and floods within cities (Berndtsson, 2010; Mentens, Raes, & Hermy, 2006).

The delivery of ecosystem services by green roofs is dependent on both plant selection and local environmental conditions (Aloisio, Palmer, Giampieri, Tuininga, & Lewis, 2017; Getter & Rowe, 2006; MacIvor, Margolis, et al., 2016; Oberndorfer et al., 2007), which in turn affects species coexistence mechanisms that depend on niche complementarity (Silvertown, 2004). Green roofs are difficult growing environments for plants because they often consist of shallow growing substrate and are exposed to high winds and full sun (Dunnett & Kingsbury, 2010; Snodgrass & Snodgrass, 2006). Additionally, many green roofs use minimal supplemental irrigation which exacerbates drought conditions (Butler & Orians, 2011; Farrell, Mitchell, Szota, Rayner, & Williams, 2012). Green roofs are also isolated and sometimes located at high elevations above ground level (Dunnett & Kingsbury, 2010). To ensure survival on green roofs, plants are generally limited to a selection of a few hardy, drought‐tolerant succulent species such as those in the genus *Sedum*, as well as *Allium* (Chives) (Butler, Butler, & Orians, 2012; Rowe, Getter, & Durhman, 2012; Snodgrass & Snodgrass, 2006).

Choosing to install simple plant communities of just a single or few species might limit the potential benefits of green roofs (Cook‐Patton & Bauerle, 2012) because complementary traits will be missed and there will be a lower probability of selecting groups of coexisting species that better utilize local space and resources. Facilitative interactions are important in stressful environments (Brooker et al., 2008) and more likely to occur in diverse communities (Mulder, Uliassi, & Doak, 2001). For example, Butler and Orians (2011) found that *Sedum* acted as a “nurse” plant for herbaceous species, thereby promoting survival and roof cooling. Manipulations of biodiversity in experimental meadows have shown that higher plant diversity is positively related to ecosystem service delivery (i.e., Cardinale et al., 2007; McGill, Enquist, Weiher, & Westoby, 2006; Tilman, Reich, & Isbell, 2012). Similarly, manipulations of plant species richness and abundance on green roofs have indicated that higher plant species diversity can promote green roof ecosystem services compared to monocultures (Lee, Williams, Sargent, Farrell, & Williams, 2014; Lundholm, MacIvor, MacDougall, & Ranalli, 2010; Madre, Vergnes, Machon, & Clergeau, 2013). In one study, Johnson, Schweinhart, and Buffam (2016) found that increasing the number of plant species in green roof plant communities improved nitrogen fixation and the contribution of green roofs to reducing urban air pollution.

Diversity in the functional traits of a plant community can also have positive impacts on a number of ecosystem services provided by green roofs (Lundholm, 2015; Lundholm, Tran, & Gebert, 2015; Van Mechelen, Van Meerbeek, Dutoit, & Hermy, 2015). This has been attributed to niche or resource partitioning among species within a designed plant community, where greater diversity results in higher usage of the available resources, thereby allowing for higher community productivity and ecosystem stability (Cadotte, Dinnage, & Tilman, 2012). However, using functional traits in a predictive way can be problematic because many ecosystem functions are ecologically complex and result from a combination of multiple traits which may not all be easy to measure, identify, or implement in practice (Cadotte, Arnillas, Livingstone, & Yasui, 2015). An alternative approach is to measure the evolutionary relationships between species (Faith, 1992; Vane‐Wright, Humphries, & Williams, 1991), which may predict functional diversity because evolutionary changes occur across multiple traits and niches (Cadotte, Cardinale, & Oakley, 2008; Gerhold, Cahill, Winter, Bartish, & Prinzing, 2015). Functional and ecological similarities are shaped by patterns of common ancestry (Cadotte, Davies, & Peres‐Neto, 2017), where species that are more closely related are likely to exhibit greater similarity in functional traits. In contrast, species that are more distantly related, therefore more phylogenetically diverse, may be less likely to share functional traits (Faith, 1992; Losos, 2008).

Phylogenetic diversity (hereafter referred to as PD) in plant communities has been considered in the implementation of ecological restoration (Hipp et al., 2015) and green infrastructure (MacIvor, Margolis, et al., 2016; MacIvor, Cadotte, Livingstone, Lundholm, & Yasui, 2016). However, very few studies have manipulated PD and demonstrated an impact on ecosystem services (Cadotte, 2013; Narwani, Matthews, Fox, & Venail, 2015). Although one study found a phylogenetic signal in plant communities for some green roof services after completion of a four‐year study, with improved roof cooling correlated with higher PD (Xie, Lundholm, & MacIvor, 2018), no study has manipulated PD experimentally to test its contribution to green roof ecosystem service delivery.

In this study, for the first time, we manipulate PD explicitly to examine impacts on green roof ecosystem service delivery, while controlling for species richness and abundance. We were interested in whether there is a link between PD and improvements to green roof cooling and rainwater management. Since tolerance of extreme environmental conditions are likely to be deeply phylogenetically conserved, and green roofs provide environmental conditions that are substantially harsher than those found at ground level, it is possible that lower PD would be important for green roof survival and performance. However, it is also possible that because we can create communities of plants known to survive on green roofs, (a) PD will increase green roof cooling because high PD communities improve productivity (e.g., Cadotte, 2013) which is linked to evapotranspiration and other mechanisms relating to green roof temperature reductions, and (b) PD will be positively correlated with the total volume of water retained by green roofs immediately after a storm event, thereby decreasing the amount of water runoff. Further, after a rain event (c) PD will be positively related to water loss in a green roof through mechanisms that do not lead to runoff (i.e., via evapotranspiration), allowing more water to be captured at the next rain event (VanWoert et al., 2005). Finally, we hypothesized that increasingly stressful and exposed site conditions as measured by building elevation was expected to lead to (d) an increase in green roof temperatures, as well as (e) greater volumes of water captured, because more exposed green roofs will dry faster, and thereby have greater carrying capacities for subsequent rain events.

## 2 MATERIALS AND METHODS

### 2.1 Study site

Eight rooftop and one ground level control sites were selected at the University of Toronto Scarborough campus in Toronto, Ontario, Canada (43°47′ N, 79°11′ W). Among rooftop sites, elevation from ground level ranged from 4.0 to 17.5 m. The ground level site was atop concrete paving stones to simulate the imperviousness of the building rooftops (Table 1 for additional site descriptions). During the study period (June–September 2015), the average monthly air temperature ranged from 18.9°C to 21.9°C and a total 380.6 mm of rainfall was recorded from the University of Toronto Scarborough weather station located on the roof of the Science building. We were only able to record data over a single season, and we acknowledge the short duration of this study.

**Table 1:** Description of sites including elevation (number of building levels), and mean values from all planting combinations mean, and maximum temperatures, the proportion of water captured (% of total supplemental irrigation), and water lost via evapotranspiration (% of water captured)

Site code	Elevation (m)	Type	Temperature (°C)			Water volume	
			Min	Mean	Max	Capture	Loss
MW	12	Roof	17.84 ± 0.32	21.55 ± 0.29	25.36 ± 0.46	0.85 ± 0.04	0.53 ± 0.07
SWG	14	Roof	18.07 ± 0.33	22.08 ± 0.31	26.51 ± 0.50	0.76 ± 0.04	0.72 ± 0.05
AA	5.75	Roof	19.25 ± 0.37	22.00 ± 0.45	25.11 ± 1.08	0.76 ± 0.07	0.57 ± 0.03
SC	4	Roof	17.92 ± 0.25	21.78 ± 0.29	25.75 ± 0.67	0.75 ± 0.05	0.61 ± 0.04
ICS	8	Roof	18.01 ± 0.40	22.32 ± 0.37	27.03 ± 0.46	0.80 ± 0.05	0.63 ± 0.08
ICM	14	Roof	19.06 ± 0.43	21.69 ± 0.46	24.09 ± 0.72	0.73 ± 0.04	0.59 ± 0.11
ICT	17.5	Roof	17.63 ± 0.37	21.65 ± 0.25	26.13 ± 0.41	0.80 ± 0.03	0.64 ± 0.04
BV	10	Roof	17.70 ± 0.35	21.70 ± 0.33	26.16 ± 0.58	0.80 ± 0.05	0.67 ± 0.06
BS	0	Con	19.48 ± 0.28	21.35 ± 0.26	23.03 ± 0.39	0.79 ± 0.06	0.57 ± 0.09

### 2.2 Green roof modules

A green roof modular array was assembled at each of the nine sites with 13 modules comprised of 42 cm × 53 cm × 15 cm wire baskets with a mesh grid of 2.5 cm × 2.5 cm. Each module was lined with a single layer of landscaping fabric, and then, a fitted piece of conventional green roof plastic drainage layer was placed on top followed by a second layer of landscaping fabric to ensure all substrate and plant material was contained within the module but that water could move through and pass out the bottom, as is the case with traditional modular green roof systems (Dunnett & Kingsbury, 2010). Commercially engineered green roof growing substrate (GroBark, Georgetown, ON) was added to each module to a depth of ~12 cm. There were two replicates of each of six planting combinations and one substrate only control at each site. Modules were set up at all sites in the same randomized pattern in a 6 × 2 arrangement with one additional module at the end of one row (total *N* = 13). All modules were rotated twice over the sampling period to account for edge effects by moving two modules from the end of one row to the opposite end of the second row and shifting the rest down.

### 2.3 Species pool, phylogeny, and plant combinations

**Figure 2:**
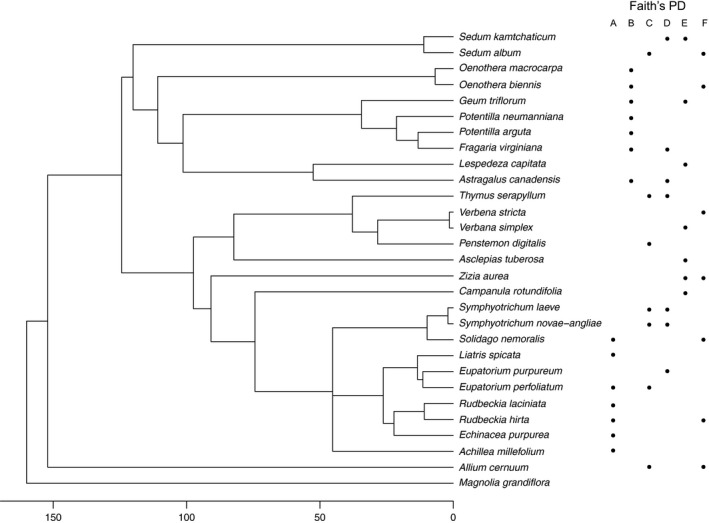
Phylogenetic tree containing all plant species that comprise the six PD combinations (Faith's normalized PD values = A: 208.48, B: 398.64, C: 610.96, D: 614.70, E: 740.60, F: 782.01)

Twenty‐eight plant species, native to Eastern North America, and three nonindigenous plant species were selected based on their success and survival on regional green roof projects (Hawke, 2015) and specifically in Toronto where our study took place (Torrance, Bass, MacIvor, & McGlade, 2013) (Figure 2). To control for species richness and abundance, each module contained 14 plants, the maximum that could be planted per module to permit ~8 cm between individuals, and so two individuals of each of seven species were represented.

To select plant combinations to represent the PD gradient, we constructed a phylogenetic tree. All species were queried in GenBank (Benson, Karsch‐Mizrachi, Lipman, Ostell, & Wheeler, 2005) for the following five commonly sequenced gene markers: *matK*,* rbcl*,* ITS1*,* ITS2*, and *5.8s*. A representative of an early diverging lineage, *Magnolia grandiflora* L.*,* was used as the outgroup species. Twenty‐four species had at least one marker found in GenBank (see Supporting Information Table S1). Species with missing gene markers were replaced with congeneric relatives (acting as proxies) that were not present in this experiment (see Supporting Information Table S1). Independent sequences were aligned using ClustalX (Larkin et al., 2007). All five sequences were concatenated into a single supermatrix using FasConCAT v1.0.pl (Kück & Meusemann, 2010). Out of 88 possible models, AIC (Akaike's Information Criterion) selected *GTR+I+ Γ* as the best‐fit nucleotide substitution model for the supermatrix, as implemented in jModelTest2 (Darriba, Taboada, Doallo, & Posada, 2012). Bayesian Inference (BI) was conducted to estimate the phylogeny using MrBayes 3.2, through the Phylemon 2.0 web server (Ronquist et al., 2012; Sánchez et al., 2011). Two independent Monte Carlo Markov Chains were run, each with three heated chains, for up to one million generations. For each separate MCMC run, every 1000 generations were sampled. Convergence was verified using Tracer 1.6 (Rambaut, Suchard, Xie, & Drummond, 2014), as indicated by a visual inspection of a stationary distribution and ESS values above 200. Nonparametric smoothing rates were performed on the BI phylogeny to obtain an ultrametric tree. Divergence times were estimated using the penalized likelihood method (Sanderson, 2002) with the chronopl function from the “ape” package (Paradis, Claude, & Strimmer, 2004) in the R statistical program v3.2.2 (R Core Team, 2015). From a cross‐validation of different lambda values, a lambda parameter value of 1000 corresponded to the cross‐validation criterion minima. The tree was then time calibrated using the *rescale* function through the “geiger” package, with the scale set to 160 million years, which is a conservative estimate for the monocot and dicot split (Chaw, Chang, Chen, & Li, 2004; Harmon, Weir, Brock, Glor, & Challenger, 2008). From the completed phylogenetic tree, six combinations of seven species were determined that formed a PD richness gradient (Figure 2). Fourteen plant species (of 28) were used in two different PD combination, but none were used in three or more combinations (see Figure 2). The two most phylogenetically distant species in the study (*Sedum* and *Allium*) were included in the treatment combinations such that two combinations had neither *Sedum* nor *Allium* (Group A and B), two had *Sedum* and not *Allium* (Group D and E), and two had both *Sedum* and *Allium* (Group C and F). We characterized the PD gradient using Faith's PD, in the package “picante” (Kembel et al., 2010).

### 2.4 Roof cooling

Daily roof surface temperatures were recorded from every module over the study using an iButton temperature logger (1‐wire Maxim, Thermochron) in a small plastic waterproof bag buried into each module just below the substrate surface. The iButtons logged temperature every 2 hr from June 26th to September 16th. From the raw temperature datasets, we calculated the daily mean, minimum, and maximum temperatures, as well as the range in daily temperatures between the minimum and maximum values. Ambient air temperatures were recorded from each site using Onset Hobo Pendant temperature data loggers set up for the duration of the study.

### 2.5 Rainwater management

The amount of rainwater captured by the module (plants and substrate) immediately following a simulated rain event was determined for all modules using a watering and weighing protocol (MacIvor & Lundholm, 2011). The protocol requires three weighing measurements from every module using an industrial freight scale (±0.005 kg) in the span of 48 hr over a nonrainy period. After each module was initially weighed (T1), three liters of water were added slowly in 1‐L intervals to each module from graduated cylinders to simulate an artificial a rain event. Thirty minutes after watering, the modules were weighed again (T2) and the difference between them was considered the amount of water captured from the rain event (Capture = T2 − T1). The water draining from the module during this period was considered the volume of water runoff. The modules were then weighed again after 48 hr (T3), resulting in the amount of water lost (Loss = T2 − T3). Water lost by T3 after T2 was assumed to be lost via evapotranspiration as all water lost before T2 was assumed to be lost via runoff.

### 2.6 Analysis

The daily minimum, mean, and maximum temperature (°C) values recorded were averaged for the full season for each module at each site, and for the ambient air temperature at each site. We tested the change in ambient conditions (minimum, maximum, and mean temperature as well as daily temperature range) with building elevation using Spearman's correlation tests. Linear mixed effect models were used to evaluate the response of the mean daily temperature in response to the PD value of the plant combination. To account for the effect of the environmental conditions, we included site as a random term and added elevation and site mean ambient air temperature as fixed terms. Similar models were used to test the response of daily minimum, maximum, and diurnal range to the same set of variables, changing only the environmental variable to ambient air minimum, maximum, and diurnal range, respectively. Site was included as a random factor and we used “lmerTest” (Kuznetsova, Brockhoff, & Christensen, 2014) to test the significance of the results, then visualized using the package “visreg” (Breheny & Burchett, 2016). All analyses were completed using the R statistical program v3.2.2 (R Core Team 2015). A similar approach using the same parameters was used to test for effects of plant community PD and elevation on rainwater capture and loss using mean ambient air temperature (°C) as the environmental variable. All residuals and random term were tested for normality using the Shapiro–Wilks test.

The analyses described above included all six plant combinations, but to evaluate the robustness of these findings, we also re‐ran these analyses with Group A and B removed (neither *Sedum* or *Allium* included in either group), Group C and F removed (both *Sedum* and *Allium* included in each group), and Group D and E removed (included *Sedum* but not *Allium*) (See Supporting Information Table S2). Results were mostly consistent among the re‐runs, and therefore, we presented the analysis of all plant combinations in the results and discussion.

## 3 RESULTS

### 3.1 Roof surface temperature

There was no significant effect of building elevation on any of the temperature parameters analyzed (*p*‐value > 0.1, see Table 2). However, nonsignificant trends point toward a positive impact of elevation on lower mean and maximum temperature, but a negative impact on minimum temperature. Elevation also increased the diurnal temperature range “DTR.” The ground level site was very exposed and so the extreme values recorded caused most of the trends attributable to elevation in this study.

**Table 2:** Pearson's correlation between ambient conditions and elevation with and without the ground level sample

	*df*	Mean	Maximum	Minimum	DTR
Including ground level					
* r*		0.486	0.461	−0.457	0.491
* p*‐value	7	0.185	0.212	0.217	0.180
Without ground level					
* r*		0.224	0.039	−0.164	0.107
* p*‐value	6	0.593	0.927	0.698	0.801

“DTR” refers to the diurnal temperature range.

**Figure 3:**
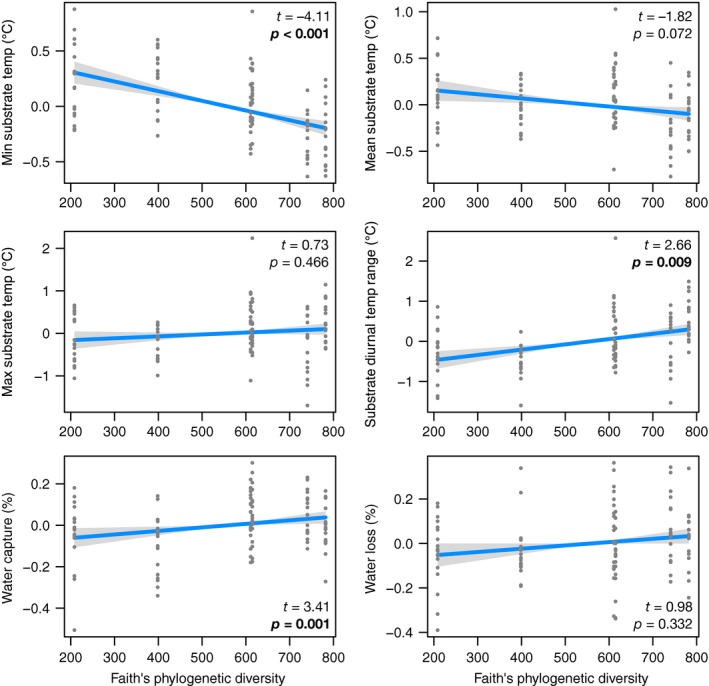
Partial effect of Faith's phylogenetic diversity (million years; Myr) on ground level temperature and water management properties

We found that increasing PD led to a significant decrease in the minimum roof surface temperature (*t* = −4.11, *p*‐value = 8.22e^−05^) and a moderately significant decrease in the mean roof surface temperature (*t* = −1.82, *p*‐value = 0.072) (Table 3, Figure 3). Increasing PD had no effect on maximum temperatures (*t* = 0.73, *p*‐value = 0.466) (Figure 3) but led to a significant increase in DTR (*t* = 2.66, *p*‐value = 0.009) (Figure 3). Most of the environmental variation was captured by the site random effect, leaving little or no variation to be explained by the elevation alone (*p*‐value > 0.1, for all temperature measures). However, ambient local conditions had a positive effect on each of the temperature variables at the module level and was significant for maximum (*t* = 4.54, *p*‐value = 0.004), and DTR (*t* = 7.04, *p*‐value = 0.0004), but not minimum or mean temperature (Table 3). Despite the significance of the sign, the amount of variance explained by PD was relatively low (Figure 3).

**Table 3:** Effect of phylogenetic diversity and ambient conditions on daily ground temperature variables and on rainwater management averaged over the growing season. Ground temperature variables include minimum, mean, maximum, and diurnal temperature range (DTR). “Elevation” is recorded as the height in meters from ground, “Ambient” is the air temperature recorded in the same site and summarized using the same function as the one used to measure the ground conditions. Rainwater management variables used mean ambient temperature as a temperature covariate. “PD” is the Faith's phylogenetic diversity of the community. Random terms represent the estimated standard deviations (*SD*) associated with the site effect and the residual of the model. The fixed terms represent the estimated effect of each independent variable and its significance level. A significance *t* test was performed with Satterthwaite approximation to determine the degrees of freedom (*p < 0.100 and ***p < 0.050). Elevation and ambient variables for temperature and water had *df* = 6, while PD had *df* = 96 for temperature and *df* = 98 for water management

	Temperature				Rainwater management	
	Minimum	Mean	Maximum	DTR	Capture	Loss
Fixed terms						
Elevation	−0.023	−0.016	−0.018	−0.019	−0.003	−0.003
Ambient	0.535	0.161	0.315***	0.435***	0.054	0.115*
PD (/100)	−0.083***	−0.035*	0.027	0.110***	0.026***	0.009
Random terms						
Site (*SD*)	0.737	0.290	0.669	1.108	0.107	0.107
Residual (*SD*)	0.350	0.336	0.615	0.710	0.135	0.160

### 3.2 Water capture and loss

There was evidence that increasing PD of the manipulated plant community led to increasing rainwater capture (*t* = 3.41, *p*‐value = 0.001) but not loss via evapotranspiration (*t* = 0.98, *p*‐value = 0.332) (Table 3, Figure 3). Mean ambient air temperature had no effect on rainwater capture (*t* = 1.05, *p*‐value = 0.335), but there was a marginal relationship with rainwater loss (*t* = 2.19, *p*‐value = 0.071). Site variability was lower than the residuals’ variability for both rainwater capture and loss, but captured most of the intersite variability, with elevation having no significant effect (capture: *t* = −0.355, *p*‐value = 0.734; loss: *t* = −0.320, *p*‐value = 0.753).

## 4 DISCUSSION

Despite the short duration of our study, we find new evidence that the manipulation of PD could improve green roof ecosystem services (Xie et al., 2018). Our data support our first hypothesis (a) that increased PD would improve green roof cooling. We found higher PD communities reduced the minimum and mean diurnal roof surface temperature but had no effect on its maximum temperature. These combined effects caused an increase in diurnal variation in roof surface temperature with increasing PD. We also found evidence to support our second hypothesis (b) that increasing PD would be correlated with the volume of water captured following a rain event, but our third hypothesis (c) was rejected as no correlation was found between PD and water loss via evapotranspiration. This could be an artifact of plant selection, as *Sedum* when mixed with herbaceous perennials are among the top performing plant communities for green roof water capture (Lundholm et al., 2010), even though it is known that *Sedum* impedes water loss from green roof substrates compared to herbaceous plants (Wolf & Lundholm, 2008). Environmental conditions (i.e., site differences) captured a significant part of the variation among sites; however, the effect of building elevation on temperature was weak and added little information, so we rejected our final two hypotheses (d) and (e). Other factors might have contributed that were not measured including wind and reflectivity of nearby windows or structures on the roof that alter local temperatures.

The reduction in minimum diurnal temperature with increasing PD may be related to an increase in productivity or photosynthetic activity, with the concomitant increase in evapotranspiration and heat transfer to the atmosphere. Greater green roof plant biomass increases the surface area available for evapotranspiration, resulting in greater water loss (MacIvor, Ranalli, & Lundholm, 2011). With a lower temperature at the end of the day, and with less water in the module, the temperature at the end of the night (when the lowest temperatures occur) will additionally be lower. Another related factor could be that higher PD communities have a more diversified canopy (Givnish, 1987), increasing the complexity of foliage and reducing gaps that allow sunlight to directly hit the substrate surface, increasing the temperature. This result may also reflect a link between PD and the reduction in the heat stored during daytime, but not to a reduction in daytime temperatures. This might explain why a reduction in mean and minimum temperatures, but not maximum temperatures, was observed. Other studies have found high plant cover to be significant for roof cooling (Speak, Rothwell, Lindley, & Smith, 2013; Takakura, Kitade, & Goto, 2000). More work is needed to specifically test the relationship between PD, evapotranspiration, plant canopy complexity, and the combined effects on thermoregulation.

An important limitation is that our modules were constructed and the data were collected during a single season. We would expect that diversity–function relationships become stronger over time as niches are filled through recruitment and that the realization of the outcomes of coexistence mechanisms would optimize resource use (Cardinale et al., 2007). Therefore, multiyear monitoring of this experimental system might yield greater differences in cover and other community‐level traits among PD treatment combinations. Further, multiyear studies would shed light on the relationship between PD and survival of green roof plant communities, a key property of any successful green roof installation.

We did not account for plant size or productivity when selecting plants for the phylogenetic analysis used to structure the different plant treatment combinations. Rather, the 28 plant species selected for this study were based on previous success (i.e., survival) on green roofs in the city and surrounding region. Practitioners do not traditionally select plant species to create maximally (or minimally) evolutionarily divergent plant combinations (i.e., high PD to improve resource partitioning), or select plants based on a phylogenetic relationship for a relevant green roof condition (i.e., low PD based on drought tolerance) (Cook‐Patton, 2015; Van Mechelen et al., 2015). More experiments are needed that manipulate species, functional, and phylogenetic diversities in designed plant communities for green infrastructure in order to better understand the ecological mechanisms that improve survival, performance, and management.

The trends reported in this study are possibly also due to the presence or absence of *Sedum* and possibly *Allium*. Harsh conditions on green roofs afford a narrow niche space where success is likely determined by environmental tolerances that are deeply phylogenetically conserved. The genus *Sedum* is not native to the Great Lakes Region, and as well, it is more drought tolerant than the other herbaceous species used in the study (Monterusso, Rowe, & Rugh, 2005; Rowe et al., 2012). The native *Allium cernuum* was also phylogenetically distant from all other plants in the study, and the genus *Allium* is perhaps the second most common green roof plant type used around the world due to its drought tolerance. We found plant mixes that contained both *Sedum* and *Allium* (C and F) or *Sedum* only (D and E) were all of relatively high PD and performed better than the plant mixes of low PD (A and B) that did not contain *Sedum* or *Allium*. Moreover, PD was not significant when plant mixes A and B were removed from analysis (Supporting Information Table S2). It is possible that the complexities of the phylogenetic methods performed provided no additional information beyond what can be interpreted taxonomically.

Difficult growing conditions on green roofs are common and often building‐ and/or region‐specific (Dunnett & Kingsbury, 2010; Rowe, 2011). As these factors may induce trait convergence regardless of the phylogenetic relation among the species, choosing plants based on functional traits to predict ecosystem service deliver under specific green roof conditions, rather than the phylogenetic distance between plant species within a community, might be more informative (MacIvor, Margolis, et al., 2016; MacIvor, Cadotte, et al., 2016). However, moving beyond the restricted set of well‐known species we have evaluated here in this study, PD can provide a first approach for practitioners to identify potential species, especially when detailed trait information for the species is unavailable, facilitating the usage of green roofs without the need for reliance on introduce nonindigenous species (i.e., *Sedum*) in cities around the world. These might include species or communities found in local habitats that exhibit similar traits or phylogenetic relationships as successful green roof plant communities (Lundholm & Walker, 2018). PD can inform which species are more likely to add to green roof ecosystem service delivery, because PD can capture processes not included in any measured functional trait. Moving forward, the plant traits that are relevant to green roof services should be tested for phylogenetic niche conservatism and distinctiveness within the pool of species currently used. This will result in a useful assessment of the feasibility of the phylogenetic approach to green roof design. If phylogenetic niche conservatism and a high degree of distinctiveness are demonstrated in at least a few relevant plant traits, then the phylogenetic approach may be applicable.

Lastly, a large proportion of the variability observed in this study was related to site differences, but not captured by elevation. Elevation and other building attributes lead to large variation in air temperature, and exposure to wind, rain, and sunlight; however, the majority of green roof research published to date are conducted on a single roof (MacIvor & Lundholm, 2011; Monterusso et al., 2005; Stovin, Vesuviano, & Kasmin, 2012). Our study demonstrates the importance of replicating green roof sites when the objectives are to link plant performance to green roof services through plant selection, as no two green roofs are the same and abiotic conditions can vary greatly (Brown & Lundholm, 2015). Often ignored in many green roof experiments is that one plant community may be optimal for a set of environmental conditions on one green roof, but incompatible with environmental conditions at another. We recommend that this roof to roof variation be evaluated in studies to determine its impact on and interaction with plant species diversity (i.e., PD) and performance. These approaches might lead to evidence for considering PD in some conditions and not others; for example, PD being important for windy, nonirrigated green roofs with shallow substrate, but not partially shaded and irrigated green roofs with deeper substrate.

### 4.1 Management implications

Here we show experimentally, and with a limited dataset, that manipulating PD in designed plant communities can improve green roof cooling and water management, when planted species richness and abundance are kept constant. Our results apply the vast literature on biodiversity effects on ecosystem services (e.g., Tilman, Isbell, & Cowles, 2014) to a human‐dominated system, where the services evaluated have clear economic and urban management implications. There is a suite of other ecosystem services that were not examined, such as pollution mitigation (Rowe, 2011), wildlife habitat (MacIvor & Ksiazek, 2015), and esthetic appreciation (Loder, 2014), all of which could potentially be influenced by manipulating PD.

An increasing number of studies aim to investigate the use of locally occurring native plant communities on green roofs (Butler et al., 2012; Heim & Lundholm, 2014; Simmons, 2015). Interpreting the phylogenetic community structure of species pools adapted to local microclimatic conditions that are similar to those experienced on green roofs, depending on spatial and temporal scale (Kraft, Cornwell, Webb, & Ackerly, 2007), could inform ecological design (Lundholm, 2006; MacIvor, Margolis, et al., 2016; MacIvor, Cadotte, et al., 2016). For example, a designed plant community could be based around one or a few key high‐performing plant species, then other species added in order to maximize the community PD. In fact, if phylogenetic information is to be useful in the process of plant selection, it has to be translated into a set of criteria that is more easily understood and user‐friendly to an average practitioner.

Understanding how evolutionary relatedness in natural communities maintains diversity and assembly can improve how biodiversity–ecosystem function relationships are measured and implemented (Gerhold et al., 2015; Gravel et al., 2012; Mouquet et al., 2012). The study of phylogenetic relationships in the service of environmental design is a new and exciting field, and a deeper understanding of phylogenetic and evolutionary mechanisms could help practitioners to broaden their set of tools used to optimize the design and management of green infrastructure. Trends that link plant community and ecosystem services are critical in interpreting best practices for green infrastructure and will support its contribution to healthy cities and to mitigate and adapt to the impacts of a changing climate.

## CONFLICT OF INTEREST

None declared.

## DATA ARCHIVING STATEMENT

GenBank accession numbers for each plant species are included in Supporting Information Table S1. The green roof temperature and rainwater collection data, and R code used for all analyses are archived on GitHub and available at this URL: https://github.com/macivorlab/C99-greenroofs.git.
