# Supplementary material for: Manipulating plant phylogenetic diversity for green roof ecosystem service delivery
Source: Evol Appl. 2018 Sep 28;11(10):2014–24. doi: 10.1111/eva.12703 (PMC6231477; doi:10.1111/eva.12703)
Supplement: Supplementary file 1 [file EVA-11-2014-s001.docx]

Table S1. Gene availability for the taxa used in the experiment. Cell codes refer to GenBank accession numbers (www.ncbi.nlm.nih.gov/genbank). 5.8s, 5.8s ribosomal RNA; ITS1 and ITS2, internal transcribed spacers 1 and 2; matk, chloroplast maturase K gene; rbcL, and ribulose-biphosphate carboxylase gene. Four congeneric species were identified (*Sedum kamtchaticum – Sedum rupestre, Sedum spurium- Sedum forsterianum, Symphyotrichum leave - Symphyotrichum pygmaeum, Verbena simplex - Verbena bracteata*).

| Species | Gene | | | | |
| --- | --- | --- | --- | --- | --- |
|  | rbcL | matK | 5.8s | ITS1 | ITS2 |
| *Achillea millefolium* | KJ204288.1 | KJ592833.1 | AY603185.1 | AY603185.1 | AY603185.1 |
| *Allium cernuum* | - | - | KC119643.1 | KC119643.1 | KC119643.1 |
| *Asclepias tuberosa* | GQ248553.1 | KJ772566.1 | - | - | - |
| *Astragalus canadensis* | - | AY386875.1 | - | U50496.1 | L10771.1 |
| *Campanula rotundifolia* | JN571981.1 | KC474263.1 | DQ304615.1 | DQ304615.1 | DQ304615.1 |
| *Echinacea purpurea* | KF613105.1 | - | EU785937.1 | EU785937.1 | EU785937.1 |
| *Eupatorium perfoliatum* | KJ841315.1 | EU749318.1 | DQ415741.1 | DQ415741.1 | DQ415741.1 |
| *Eupatorium purpureum* | KF724260.1 | - | FJ395159.1 | FJ395159.1 | FJ395159.1 |
| *Fragaria virginiana* | JX848523.1 | KJ592952.1 | GQ476750.1 | GQ476750.1 | GQ476750.1 |
| *Geum triflorum* | JX848525.1 | - | AJ302344.1 | AJ302344.1 | AJ302344.1 |
| *Lespedeza capitata* | KJ773621.1 | KJ772888.1 | - | - | - |
| *Liatris spicata* | HQ416177.1 | HQ416287.1 | HQ416383.1 | HQ416383.1 | HQ416383.1 |
| *Magnolia grandiflora* | GQ120447.1 | AM889723.1 | - | - | - |
| *Oenothera biennis* | KM218350.1 | HQ593370.1 | EU592030.1 | EU592030.1 | EU592030.1 |
| *Oenothera macrocarpa* | AM235671.1 | - | AJ620540.1 | AJ620540.1 | AJ620540.1 |
| *Penstemon digitalis* | HQ590202.1 | - | DQ471681.1 | - | DQ471681.1 |
| *Potentilla arguta* | JX848522.1 | HQ593397.1 | U90787.1 | U90787.1 | U90787.1 |
| *Potentilla neumanniana* | JN893739.1 | JN896181.1 | - | FN666607.1 | FN666607.1 |
| *Rudbeckia hirta* | AY215173.1 | AY215856.1 | KT179623.1 | U72811.1 | U74407.1 |
| *Rudbeckia laciniata* | KJ773848.1 | KJ773099.1 | - | U72797.1 | U72405.1 |
| *Sedum kamtchaticum* | KJ204405.1 | - | KJ884130.1 | KJ884130.1 | KJ884130.1 |
| *Sedum spurium* | KF997422.1 | KJ204538.1 | KJ884125.1 | KJ884125.1 | KJ884125.1 |
| *Solidago nemoralis* | KJ593689.1 | KJ593107.1 | EU125357.1 | EU125357.1 | EU125357.1 |
| *Symphyotrichum laeve* | KC484189.1 | KC475967.1 | EU200231.1 | EU200231.1 | EU200231.1 |
| *Symphyotrichum novae-angliae* | GU817740.1 | KJ593119.1 | JQ360398.1 | JQ360398.1 | JQ360398.1 |
| *Thymus serapyllum* | KF997486.1 | AY840173.1 | EU796890.1 | EU796890.1 | EU796890.1 |
| *Verbena simplex* | HQ384875.1 | HQ384506.1 | FJ867408.1 | FJ867408.1 | FJ867408.1 |
| *Verbena stricta* | HQ590319.1 | HQ593486.1 | - | EU791336.1 | - |
| *Zizia aurea* | KT178117.1 | KT176598.1 | KT179631.1 | AH003554.1 | AH003554.1 |
