# Supplementary material for: Manipulating plant phylogenetic diversity for green roof ecosystem service delivery
Source: Evol Appl. 2018 Sep 28;11(10):2014–24. doi: 10.1111/eva.12703 (PMC6231477; doi:10.1111/eva.12703)
Supplement: Supplementary file 2 [file EVA-11-2014-s002.docx]

**Table S2.** Effect of phylogenetic diversity and ambient conditions on daily ground temperature (°C) variables and on rainwater management averaged over the growing season. As in Table 3, ground temperature variables include minimum, mean, maximum and diurnal temperature range (DTR). ‘Elevation’ is recorded as the height in meters from ground, ‘Ambient’ is the air temperature (°C) recorded in the same site and summarized using the same function as the one used to measure the ground conditions. Rainwater management variables used mean ambient temperature as a temperature covariate. ‘PD’ is the Faith’s phylogenetic diversity of the community (million years, rescaled to 100 million years). Random terms represent the estimated standard deviations (sd) associated with the site effect and the residual of the model. The fixed terms represent the estimated effect of each independent variable and its significance level. A significance t-test was performed with Satterthwaite approximation to determine the degrees of freedom (*: *p* < 0.100 and ***: *p* < 0.050). Elevation and ambient variables for temperature and water had df=6, while PD had df=96 for temperature and df=98 for water management. Models analyzed included ‘All’ which was all six treatments (A-F). Group A and B did not include *Sedum* or *Allium*. Group C and F included both *Sedum* and *Allium*, and Group D and E included *Sedum*, but not *Allium*. Bolded values between models were those that were the same as when ‘All’ treatments were included.

|  | | | Temperature | | | | Rainwater Management | |
| --- | --- | --- | --- | --- | --- | --- | --- | --- |
| **Model** |  | | Min | Mean | Max | DTR | Capture | Loss |
| All | Fixed | Elevation | -0.023 | -0.016 | -0.018 | -0.019 | -0.003 | -0.003 |
|  |  | Ambient | 0.535 | 0.161 | **0.315***** | **0.435***** | 0.054 | **0.115*** |
|  |  | PD (/100) | **-0.083***** | **-**0.035* | 0.027 | **0.110***** | **0.026***** | 0.009 |
|  | Random | Site (sd) | 0.737 | 0.290 | 0.669 | 1.108 | 0.107 | 0.107 |
|  |  | Residual (sd) | 0.350 | 0.336 | 0.615 | 0.710 | 0.135 | 0.160 |
| A + B removed | Fixed | Elevation | -0.025 | -0.023 | -0.028 | -0.029 | -0.006 | -0.004 |
|  |  | Ambient | 0.547* | 0.184 | **0.319***** | **0.443***** | 0.059 | **0.130***** |
|  |  | PD (/100) | **-**0.074 | **-**0.026 | -0.184* | -**0.259***** | 0.022 | -0.038 |
|  | Random | Site (sd) | 0.713 | 0.224 | 0.644 | 1.087 | 0.090 | 0.101 |
|  |  | Residual (sd) | 0.340 | 0.360 | 0.650 | 0.710 | 0.121 | 0.165 |
| C + F removed | Fixed | Elevation | -0.027 | -0.012 | -0.005 | -0.004 | -0.001 | -0.002 |
|  |  | Ambient | 0.549 | 0.130 | **0.304***** | **0.432***** | 0.042 | 0.082 |
|  |  | PD (/100) | **-0.139***** | **-0.083***** | 0.005 | **0.134***** | 0.024*** | **0.024***** |
|  | Random | Site (sd) | 0.779 | 0.285 | 0.715 | 1.204 | 0.107 | 0.104 |
|  |  | Residual (sd) | 0.318 | 0.305 | 0.582 | 0.671 | 0.137 | 0.145 |
| D + E removed | Fixed | Elevation | -0.017 | -0.014 | -0.021 | -0.027 | -0.002 | -0.003 |
|  |  | Ambient | 0.513 | 0.171 | **0.322***** | **0.432***** | 0.061 | **0.133***** |
|  |  | PD (/100) | -0.037 | 0.011 | 0.072* | **0.108***** | **0.027***** | -0.002 |
|  | Random | Site (sd) | 0.719 | 0.345 | 0.644 | 1.035 | 0.119 | 0.102 |
|  |  | Residual (sd) | 0.330 | 0.312 | 0.593 | 0.697 | 0.147 | 0.167 |
